# Adult-Onset Immunoglobulin A Vasculitis Following Hemodialysis Treatment: An Unusual Presentation

**DOI:** 10.7759/cureus.34984

**Published:** 2023-02-14

**Authors:** Barbara S Suening, Kylee Arthurs, Alexandra K Mathis, Karina Doucet, Chamonix Kinimaka

**Affiliations:** 1 Medicine, Edward Via College of Osteopathic Medicine, Spartanburg, USA; 2 Medicine, Edward Via College of Osteopathic Medicine, Auburn, USA; 3 Family Medicine, Orange Park Medical Center, Orange Park, USA

**Keywords:** maintenance hemodialysis, end stage renal disease (esrd), henoch schönlein purpura, adult iga vasculitis, palpable purpura

## Abstract

Immunoglobulin A (IgA) vasculitis, formerly known as Henoch-Schönlein purpura (HSP), is a small vessel vasculitis due to perivascular deposition of dominant IgA immune complexes. It classically presents with symptoms such as palpable purpura, abdominal pain, kidney dysfunction, and joint pain. It most commonly affects children less than 10 years old. We present the case of a 53-year-old male who developed purpuric rashes a few hours after receiving hemodialysis. Initially, the lesions were localized to his legs and buttocks. They continued to spread over his back, abdomen, and arms. He experienced joint pain in both of his wrists, as well as abdominal tenderness. Labs revealed elevated IgA levels: 422 mg/dL (normal: 61 - 356 mg/dL). C3, C4, and antinuclear antibody (ANA) levels were within normal limits. Oral prednisone and topical diphenhydramine resulted in significant improvement in his symptoms. To our knowledge, there are only five reports documenting the occurrence of HSP in adults undergoing hemodialysis. Although HSP is a rare finding in adults, recognition of the disease is important as it can cause significant morbidity and mortality if left untreated.

## Introduction

Immunoglobulin A (IgA) vasculitis, also known as Henoch-Schönlein purpura (HSP), is a type of vasculitis that is characterized by the deposition of IgA immune complexes in blood vessels. HSP is one of the most common types of vasculitides in children, typically affecting those between three and ten years of age [1]. It classically presents as a tetrad whose major manifestations include nonthrombocytopenic purpura, abdominal pain, renal dysfunction, and arthralgias. The lesions tend to present in dependent areas such as the legs and buttocks but can also appear in other parts of the body such as the trunk, arms, and face [1]. The immune deposits, compromising vessel wall integrity, lead to leakage of serum into the surrounding tissue. The extravasated blood results in palpable petechiae and/or purpura. They may be present with swelling and pain in affected areas. Abdominal discomfort is often described as a colicky feeling and may be accompanied by nausea, vomiting, and diarrhea [2]. Renal involvement tends to present more often in adults than in children and can manifest as hematuria, azotemia, and proteinuria [2-4]. In severe cases, it may lead to renal failure. Unlike in children, male adults are more commonly affected than female adults with HSP [4].

HSP is believed to be triggered by an immune system response to infections, medications, or an underlying health condition [2,4]. Although the exact pathogenesis remains unknown, it commonly follows upper respiratory infections and is more commonly seen in the winter months, January through March [2]. HSP may also occur in outbreaks, with several episodes occurring within a short period of time in a particular area. Other possible symptoms of HSP include swelling, joint deformities, coughing, and difficulty breathing [5]. HSP can also rarely cause chest pain, as well as neurological symptoms such as headaches, stroke, and seizures [6]. The severity and specificity of the symptoms can vary widely among patients, and not everyone with the condition will experience all of the symptoms described above. 

Biopsy of the skin is useful for distinguishing HSP from other autoimmune conditions. Pathology findings would demonstrate IgA deposition in both lesional and perilesional skin [7]. Serum IgA, C3, and C4 levels may be elevated, however, C3 and C4 can also be normal and are therefore less reliable markers [8,9]. Management includes supportive care measures such as pain management, hydration, and bed rest. Naproxen and prednisone have been reported to alleviate abdominal pain [10]. In severe cases, hospitalization may be required.

Overall the prognosis for individuals with IgA vasculitis is generally favorable if properly addressed [10]. Recurrence, which occurs in about a third of cases tends to be less severe than the original outbreak [11]. In rare circumstances, HSP can lead to serious complications such as kidney failure which can be life-threatening if not treated promptly. Therefore, although HSP is less commonly seen in adults, its recognition is significant so patients may receive appropriate medical attention in a timely manner.

## Case presentation

A 53-year-old male presented to the emergency department with the chief complaint of a painful and pruritic rash that began hours after receiving hemodialysis treatment. It started on his right leg and spread to his legs and buttocks. By the evening, his back, abdomen, and arms were covered in similar lesions. The pain was constant, progressive, 7/10, and worsened with movement. There was associated flank pain, joint pain affecting his wrists and fingers bilaterally, and numbness and tingling in his legs. His right lower extremity was warm and swollen. He admitted to having shortness of breath and non-productive coughing. He denied experiencing a similar event in the past, as well as any precipitating factors for this episode to occur. He denied fevers, chills, nausea, vomiting, recent sick contacts, changes to diet, insect or animal bites, new medications, changes to medications, or a history of HIV and hepatitis.

He had a history of asthma, congestive heart failure, diabetes mellitus type 2, end-stage renal disease (hemodialysis on Monday, Wednesday, and Friday), and hypertension. He had a left below-the-knee amputation the year prior, as well as two right metatarsal amputations. His family history was significant for cancer. He denied the use of alcohol, recreational drugs, and ever smoking. He was allergic to trimethoprim/sulfamethoxazole. His medications included ticagrelor, nifedipine, gabapentin, carvedilol, aspirin, hydralazine, and atorvastatin.

One month prior, he was admitted for peritonitis and completed antibiotic treatment. During that time, he was unable to operate his peritoneal dialysis circuit due to a catheter malfunction. As a result, he was unable to dialyze for four days. He experienced nausea, weakness, non-bloody vomiting, periumbilical pain, abdominal distention, and chills. He was temporarily transitioned to hemodialysis with plans of returning to peritoneal dialysis after the nonfunctional device was addressed with surgical revision. His symptoms resolved with hemodialysis. He had been in his usual state of health until the rashes appeared two weeks later.

**Figure 1:**
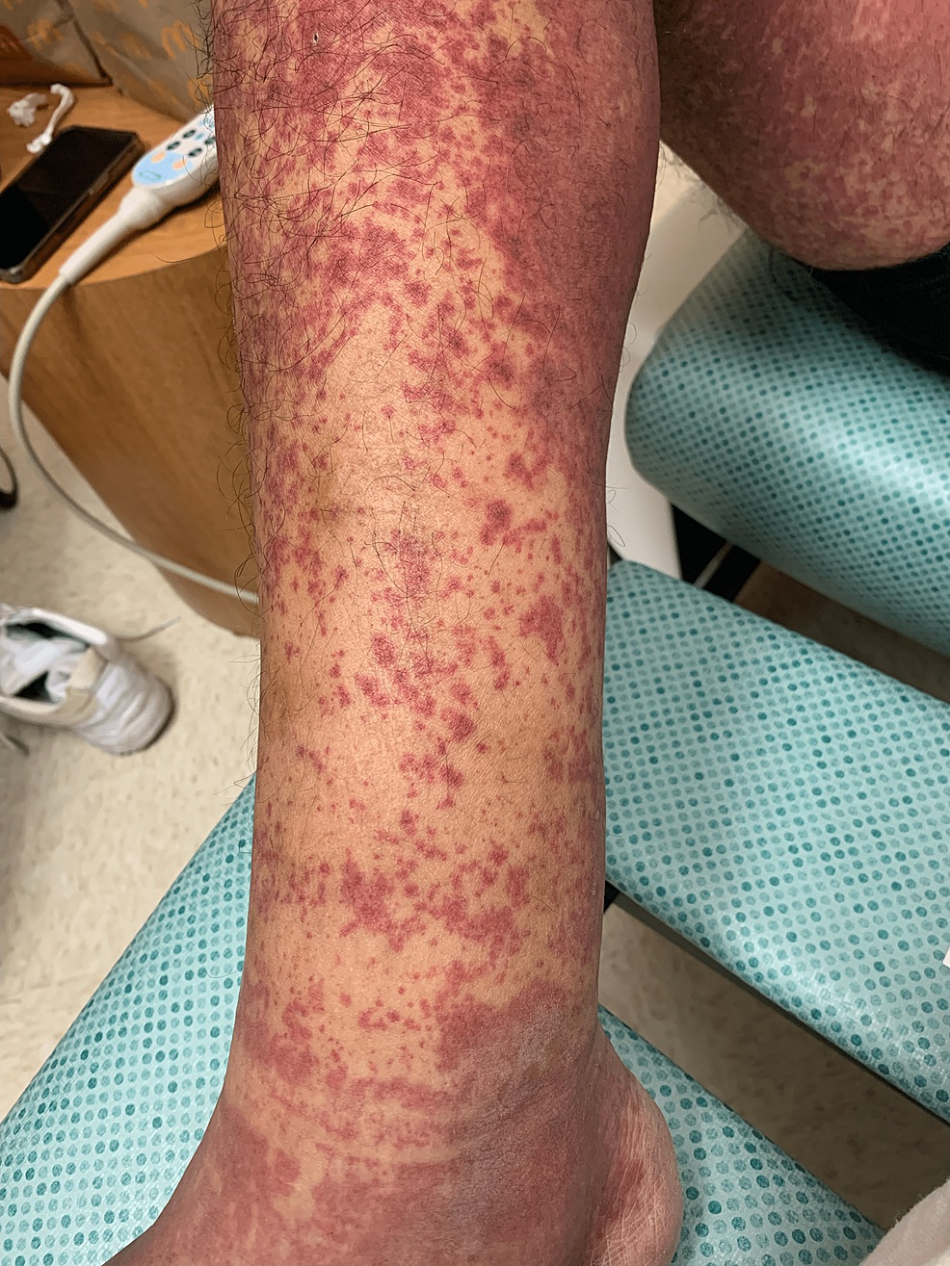
Palpable purpura diffusely spread throughout the right lower extremity

Overall the patient was in no acute distress. He was afebrile and his vital signs were within normal limits. On physical exam, his abdomen was tender to palpation and revealed the right upper quadrant port, which was not being used, without signs of infection. Costovertebral angle tenderness was positive bilaterally with associated midline lumbar spine tenderness. The right lower extremity was warm and revealed 2+ pitting edema. The skin was intact with palpable purpura diffusely spread throughout the patient’s upper extremities, lower extremities, abdomen, back, and buttocks (Figure 1). There were no open wounds, ulcers, discharge, scaling, or lymphadenopathy noted.

Coagulation studies revealed intact hemostasis: prothrombin time (PT) 11.4 sec (normal: 10.2-12.9 sec), international normalized ratio (INR) 1 (<1.1), partial thromboplastin time (PTT) 31 sec (normal: 25.1-36.6 sec). Comprehensive metabolic panel and complete blood count are listed in Table 1 and Table 2, respectively. Hepatitis panel from the previous admission was negative.

**Table 1:** Comprehensive metabolic profile BUN: blood urea nitrogen; GFR: glomerular filtration rate; AST: aspartate aminotransferase; ALT: alanine transaminase

Test	Patient’s Range	Normal Range
BUN	21 mg/dL	9-23 mg/dL
Creatinine	4.84 mg/dL	0.55-1.02 mg/dL
Estimated GFR	<15 mL/min	Above 60 mL/min
Glucose	122 mg/dL	74-106 mg/dL
Total Bilirubin	0.3 mg/dL	0.3-1.2 mg/dL
AST	20 U/L	0-34 U/L
ALT	12 U/L	10-49 U/L
Alkaline Phosphatase	115 U/L	46-116 U/L
C-Reactive Protein	57 mg/L	<10 mg/L
Total Protein	8.7 g/dL	5.7-8.2 g/dL
Albumin	4.4 g/dL	3.2-4.8 g/dL

**Table 2:** Complete blood count WBC: white blood cell; MCV: mean corpuscular volume; ESR: erythrocyte sedimentation rate

Test	Patient’s Range	Normal Range
WBC	9.0 x 10^3^/uL	4.0-10.5 x10^3^/uL
Hemoglobin	9.3 g/dL	13.7-17.5 g/dL
MCV	87.6 fl	79.0-92.2 fl
Platelet Count	422 x 10^3^/uL	150-400 x10^3^/uL
ESR	88 mm/hr	0-10 mm/hr

Hydralazine was temporarily held due to its known cause of skin reactions. Lab studies revealed elevated IgA levels: 422 mg/dL (normal: 61 - 356 mg/dL). C3, C4, and antinuclear antibody (ANA) levels were within normal levels. Direct Coombs test, HIV, and P/C-antineutrophil cytoplasmic antibodies (ANCA) results were insignificant. Urinalysis and blood cultures were negative. Dermatology was unavailable to biopsy lesions. Differential diagnoses included drug-induced vasculitis, ANCA-associated vasculitis, IgA vasculitis, and systemic lupus erythematosus. Based on clinical presentation and elevated IgA levels, a diagnosis of IgA vasculitis was made. Hydralazine was resumed. Prednisone 60mg QD and administration of topical diphenhydramine led to significant resolution of the patient’s rashes and arthralgias. Doppler ultrasound of right lower extremity was negative for deep venous thrombosis. On day four of admission, the patient was stable with marked improvement of his symptoms. He was discharged with methylprednisolone, instructed to continue hemodialysis per the usual schedule, and to follow up with his primary care provider for repeat labs in three days. Attempts were made to follow on his progression and the results of the repeat labs. The patient however was lost to follow-up, and thus the information could not be obtained.

## Discussion

Certain medications may cause inflammation of the blood vessels, leading to symptoms similar to that of HSP. Hydralazine is a medication used to treat hypertension and unfortunately, it is also known to cause a number of side effects involving the skin such as hives, rashes, and purpura [12]. During our patient’s admission, we decided to temporarily hold hydralazine in the event his symptoms may have been drug-induced. However, due to the elevated IgA levels, in addition to his overall clinical picture, a diagnosis of IgA vasculitis was made. Hydralazine was resumed without issues.

The development of HSP in the setting of hemodialysis, however, is an uncommon finding [13]. Some evidence suggests hemodialysis may be a risk factor for HSP due to its disruption of the immune system. The process involves an extracorporeal machine to circulate and filter blood, as well as frequent venipuncture, predisposing the patient to an increased risk of infection and immune system dysregulation [14]. Another study found reduced platelet activation in chronic hemodialysis patients. This results in mild degrees of thrombocytopenia which may be associated with the classic petechial rashes noted in HSP [15]. In typical patients, hemodialysis would transiently decrease platelets in the first hour but normalize by the end of treatment. This was not the case for our patient due to his elevated platelet count on admission, as seen in Table 2. Overall, the relationship between hemodialysis and HSP is not fully understood, and more research is needed to clarify this relationship.

## Conclusions

IgA vasculitis, otherwise known as HSP, is an uncommon finding in adults, especially in the setting of hemodialysis. The condition is most commonly diagnosed in childhood, however, it can occur at any age. Consideration of HSP in adults undergoing hemodialysis is important as it can cause significant morbidity and mortality from organ damage if left untreated.

## References

[1] Roberts PF, Waller TA, Brinker TM, Riffe IZ, Sayre JW, Bratton RL (2007). Henoch-Schönlein purpura: a review article. South Med J.

[2] Pillebout E, Sunderkötter C (2021). IgA vasculitis. Semin Immunopathol.

[3] Reamy BV, Williams PM, Lindsay TJ (2009). Henoch-Schönlein purpura. Am Fam Physician.

[4] García-Porrúa C, Calviño MC, Llorca J, Couselo JM, González-Gay MA (2002). Henoch-Schönlein purpura in children and adults: clinical differences in a defined population. Semin Arthritis Rheum.

[5] Bose S, Pathireddy S, Baradhi KM, Aeddula NR (2019). Seizures, renal failure and acute respiratory failure: not your typical case of Henoch-Schonlein purpura. BMJ Case Rep.

[6] Sundriyal D, Grupta BB, Sharma B, Chawla MPS (2013). Cardiac involvement in Henoch-Schönlein purpura. J Indian Acad Dent.

[7] Van Hale HM, Gibson LE, Schroeter AL (1986). Henoch-Schönlein vasculitis: direct immunofluorescence study of uninvolved skin. J Am Acad Dermatol.

[8] Mayer-Hain S, Gebhardt K, Neufeld M (2022). Systemic activation of neutrophils by immune complexes is critical to IgA vasculitis. J Immunol.

[9] Kawana S, Nishiyama S (1992). Serum SC5b-9 (terminal complement complex) level, a sensitive indicator of disease activity in patients with Henoch-Schönlein purpura. Dermatology.

[10] Dalpiaz A, Schwamb R, Miao Y, Gonka J, Walzter W, Khan SA (2015). Urological manifestations of Henoch-Schonlein purpura: a review. Curr Urol.

[11] McClain DM, Maino K, Dwyer TX (2006). Henoch-Schönlein purpura in an adult Filipino man: a case report and literature review. Cutis.

[12] Misra DP, Patro P, Sharma A (2019). Drug-induced vasculitis. Indian J Rheumatol.

[13] Chitralli DK, Churchill BM, Patri P, Puttegowda D (2020). IgA vasculitis in a patient on dialysis. Asian J Sci Res.

[14] Gao JJ, Wei JM, Gao YH, Li S, Na Y (2014). Central venous catheter infection-induced Henoch-Schönlein purpura in a patient on hemodialysis. Ren Fail.

[15] Daugirdas JT, Bernardo AA (2012). Hemodialysis effect on platelet count and function and hemodialysis-associated thrombocytopenia. Kidney Int.

